# Incidental diagnosis of pulmonary cryptococcosis by rebiopsy for epidermal growth factor receptor T790M mutation: A case report

**DOI:** 10.1111/1759-7714.14753

**Published:** 2022-11-30

**Authors:** Hiroshi Kobe, Shinya Yokoe, Tadashi Ishida

**Affiliations:** ^1^ Department of Respiratory Medicine, Ohara Healthcare Foundation Kurashiki Central Hospital Okayama Japan; ^2^ Division of Respiratory Medicine Department of Internal Medicine Shiga University of Medical Science Otsu Japan

**Keywords:** cryptococcosis, epidermal growth factor receptor, lung cancer, rebiopsy, ruxolitinib

## Abstract

Cryptococcosis is an invasive fungal infection that can occur in cancer patients. A case of pulmonary cryptococcosis in a patient treated with erlotinib + ramucirumab for epidermal growth factor receptor (EGFR) L858R point mutation‐positive non‐small cell lung cancer is presented. During chemotherapy, a new pulmonary nodule was found and considered progressive disease. Examination of the biopsy specimen taken to identify *EGFR* T790M mutation incidentally led to the diagnosis of pulmonary cryptococcosis. Three months after taking fluconazole, chest computed tomography showed that the pulmonary nodule had shrunk. New pulmonary nodules during lung cancer treatment require careful attention, not only because of disease progression, but also because of the possibility of infection in an immunocompromised host.

## Graphical abstract

During chemotherapy for *EGFR* mutation positive lung cancer, a new pulmonary nodule was found and considered progressive disease. Examination of the biopsy specimen taken to identify EGFR T790M mutation incidentally led to the diagnosis of pulmonary cryptococcosis.

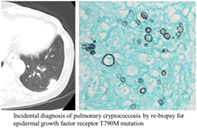

## INTRODUCTION

Epidermal growth factor receptor (*EGFR*) mutation is one of the most common oncogenic driver mutations in non‐small cell lung cancer. If the initial EGFR‐tyrosine kinase inhibitor treatment fails, repeated tumor biopsy is recommended to determine the subsequent treatment plan. The existence of *EGFR* T790M mutations is crucial for using osimertinib.^1^ The incidence of cryptococcosis has increased over the past 50 years,^2^ and pulmonary cryptococcosis mimicking lung cancer has been reported.^3^ However, it is rare that it be diagnosed incidentally on rebiopsy to search for driver mutations. A case of asymptomatic pulmonary cryptococcosis diagnosed incidentally by lung biopsy to search for T790M mutations is described.

## CASE REPORT

**FIGURE 1:**
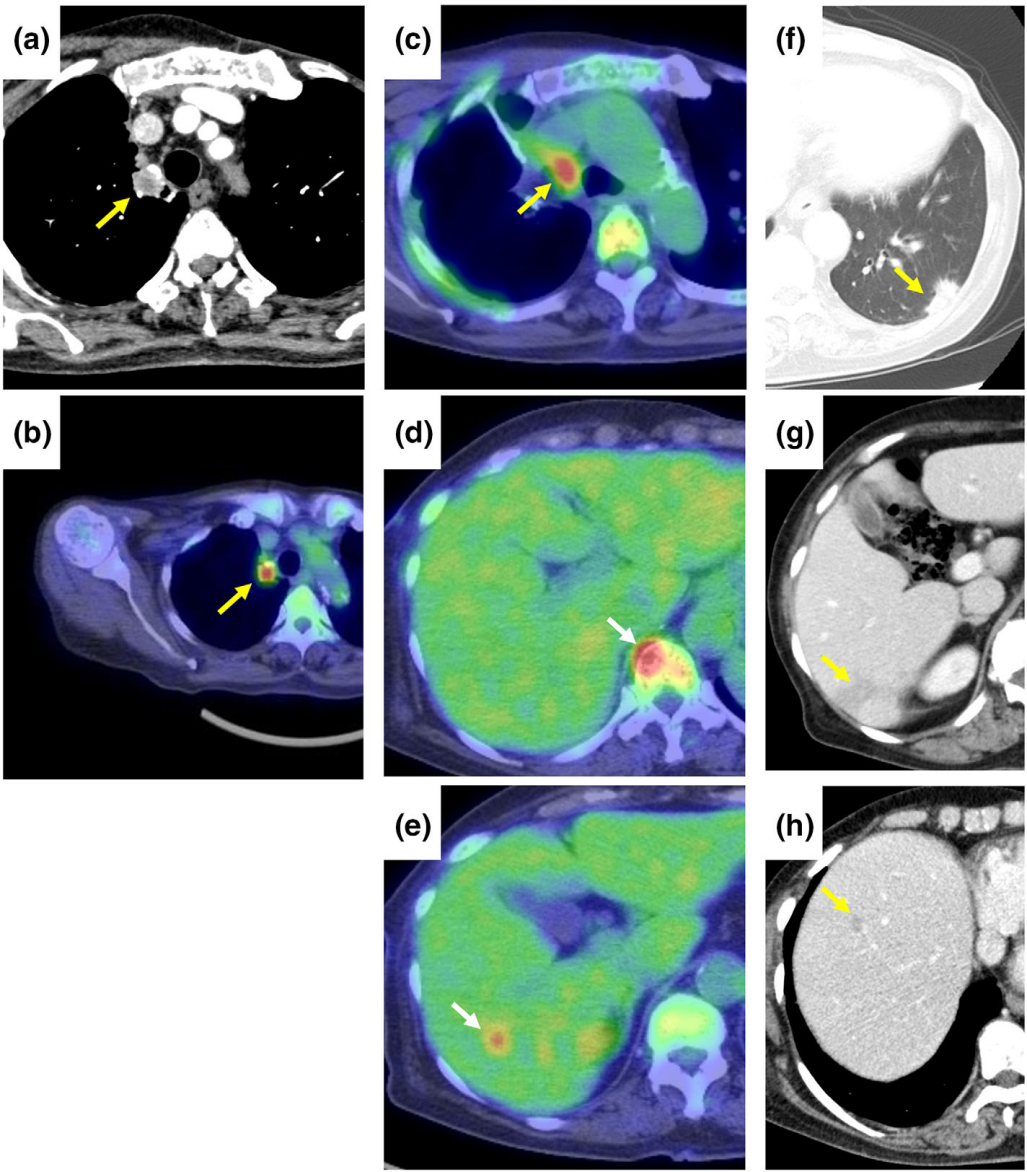
(a) and (b) Before right upper lobectomy. (a) Chest computed tomography (CT) with a 15.6‐mm nodule (arrow) localized in the right upper lobe. (b) Fluorodeoxyglucose‐positron emission tomography (FDG‐PET) CT with a nodule (arrow) localized in the right upper lobe without distant metastasis. (c)–(e) Postoperative FDG‐PET CT. FDG uptake is seen in mediastinal lymph nodes (c), bone (d), and liver (e). (f)–(h) CT after erlotinib and ramucirumab therapy. A new pulmonary lesion (f) and some liver lesions (g) and (h) are seen

**FIGURE 2:**
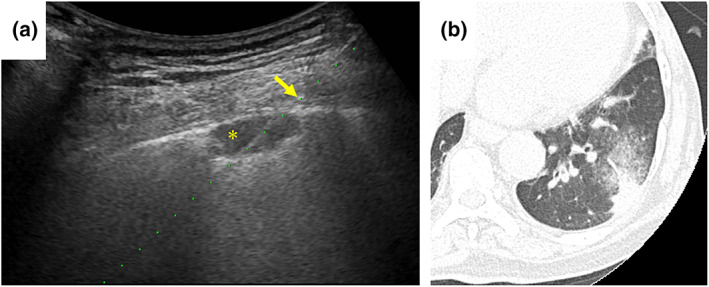
(a) Echo‐guided percutaneous needle biopsy was performed. 18‐G needle (arrow). A subpleural hypoechoic mass (asterisk). (b) After the biopsy, computed tomography (CT) shows no pneumothorax, hemothorax, or air emboli, but a localized ground‐glass opacity is seen around the mass.

**FIGURE 3:**
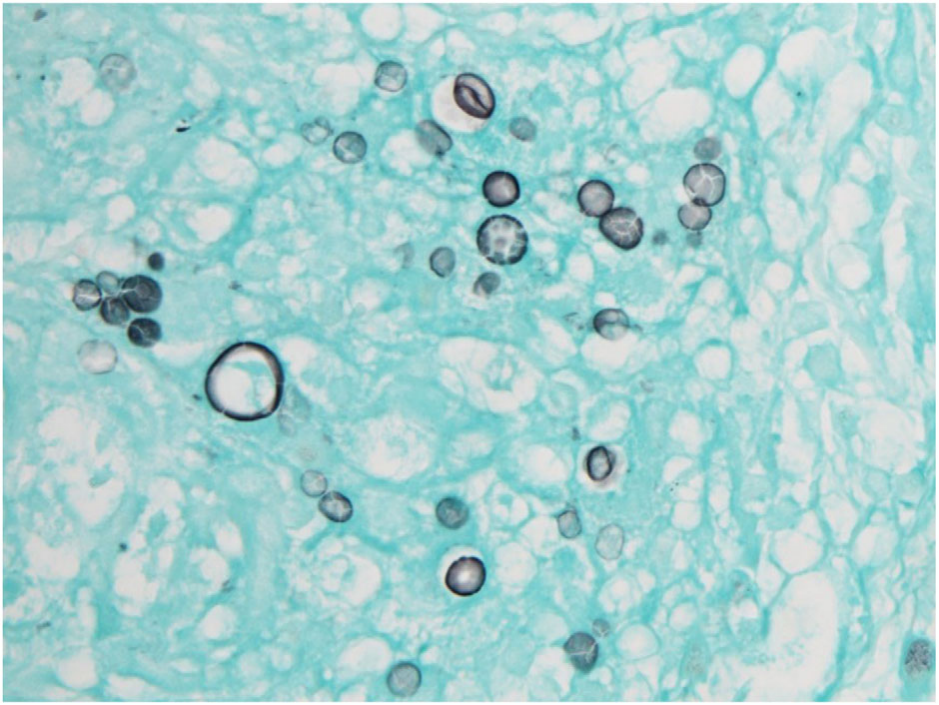
Lung tissue from the echo‐guided percutaneous needle biopsy. Grocott stain shows pulmonary cryptococcosis.

**FIGURE 4:**
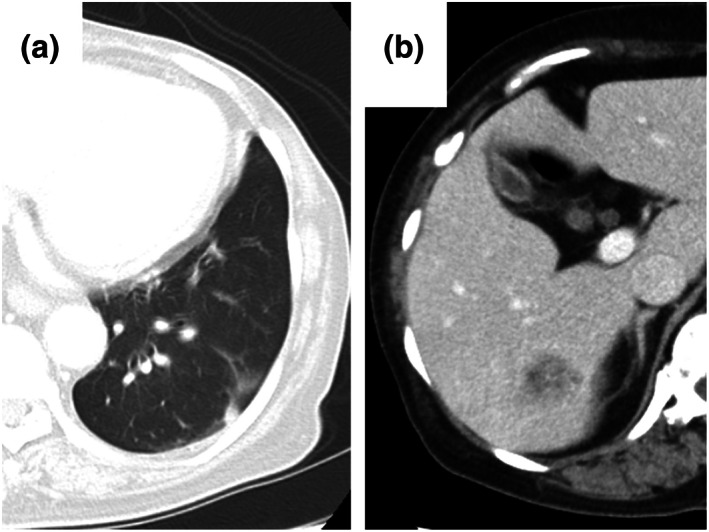
Three months after the start of fluconazole, with no chemotherapy administered for the lung cancer during this period. (a) The lung nodules have shrunk and (b) the liver metastases have enlarged.

A 77‐year‐old woman underwent resection of right upper lobe non‐small cell lung cancer (NSCLC) a year earlier (Figure 1a,b ). She had been treated with ruxolitinib for myelofibrosis 2 years earlier. The NSCLC recurred with multiple lymph node, bone, and liver metastases 9 months earlier (Figure 1c–e), and L858R point mutation was identified. Erlotinib and ramucirumab were administered as initial chemotherapy. After a partial response for 9 months, follow‐up chest‐abdomen computed tomography (CT) showed a new pulmonary lesion in the left lower lobe and liver (Figure 1f–h). The patient was admitted to hospital for echo‐guided percutaneous needle biopsy of the pulmonary lesion to search for a newly expressing *EGFR* T790M mutation. The pulmonary lesion was along the pleura and considered difficult to diagnose by a bronchoscopic approach. On admission, blood pressure was 153/90 mmHg, temperature 36.7°C, pulse 57 beats/min, respiratory rate 12 breaths/min, and oxygen saturation 99% on room air. On physical examination, the neck was supple, and there were no lung crackles, no cardiac murmurs, and no lower extremity edema. She denied cough, chest pain, dyspnea, headache, or confusion. On laboratory examination, C‐reactive protein was 0.06 (reference range, 0–0.14) mg/dl, HbA1c 6.7% (4.6%–6.2%), hemoglobin 10.5 (11.6–14.8) g/dl, white blood cells 6500/μl (3300–8600/μl), platelets 29.6 × 10^4^/μl (16.0–36.0 × 10^4^/μl), and carcinoembryonic antigen 104.9 (≤5.0) ng/ml. Head CT showed no findings of meningitis or brain metastases. The patient was placed in the prone position, and echo‐guided percutaneous needle biopsy of the pulmonary lesion was performed. A subpleural hypoechoic mass was observed (Figure 2a). One specimen was obtained for this using an 18‐G needle. After puncture, she developed cough and dyspnea, and sputum with fresh blood appeared. CT showed no pneumothorax, hemothorax, or air emboli, but a localized ground‐glass opacity was seen around the mass (Figure 2b), which was judged to be due to hemoptysis after puncture. One biopsy was performed. She was discharged from the hospital as scheduled with no evidence of pneumothorax on chest X‐ray the next day. Fungi showing positive Grocott staining confirmed pulmonary cryptococcosis (Figure 3). Serum and cerebrospinal fluid cryptococcal glucuronoxylomannan antigens were negative. Cerebrospinal fluid cytology and culture excluded cryptococcal meningitis, and fluconazole was given. Three months after having taken fluconazole, chest–abdomen CT showed that the left lower pulmonary lesion had shrunk (Figure 4a), and the liver lesions had enlarged (Figure 4b). The liver lesions were diagnosed as metastatic liver tumors. Since her general condition was deteriorating, and there was a high risk that chemotherapy would not be possible in the event of an adverse event due to liver, bone, or lymph node metastasis biopsy, liquid biopsy was the first priority. A liquid biopsy confirmed that she was negative for EGFR T790M mutation. Carboplatin and pemetrexed were administered.

## DISCUSSION

Cryptococcosis, most often caused by *Cryptococcus neoformans* and *Cryptococcus gattii*, is an invasive fungal infection that has increased dramatically over the past 50 years as new populations of immunocompromised hosts have emerged related to HIV, malignancies and their treatment, organ transplantation, and the increasing use of immunomodulating treatments.^2^

Ruxolitinib, an inhibitor of Janus kinases 1 and 2, suppresses the immune system and potentially increases infections. Although there have been reports of of the occurrence of cryptococcosis while the patient was taking ruxolitinib,^4^ according to a recent meta‐analysis,^5^ ruxolitinib for myeloproliferative neoplasms increased the risk of herpes zoster infection, but there have been insufficient studies to determine the effects of ruxolitinib on the risk of overall infection. Moreover, pulmonary cryptococcosis has been reported to result in consolidation mimicking lung cancer.^3^ In the present case, there was a new lung lesion that was not progression of lung cancer, but pulmonary cryptococcosis in a patient who had been treated with ruxolitinib and erlotinib + ramucirumab. Cryptococcal infections are caused by inhalation of the fungus, either in the form of yeast or as basidiospores from an environmental source. Infections of the central nervous system and the lower respiratory tract are the most common manifestation.^6^ The symptoms of pulmonary cryptococcosis are nonspecific, including fever, cough, dyspnea, night sweats, malaise, chest pain, and, rarely, hemoptysis. Since the patient had none of the above symptoms, and serum cryptococcal glucuronoxylomannan antigens were negative, it was impossible to suspect pulmonary cryptococcosis clinically. Lung cancer treatment is now very advanced, suggesting the usefulness of rebiopsy in non‐*EGFR* T790M mutation‐positive cases to administer osimertinib.^7^ With the current advances in cancer drug therapy, some patients present with multiple malignancies. We should keep in mind the possibility of unexpected complications due to multiple anticancer treatments.

## AUTHOR CONTRIBUTIONS

**HK:** writing ‐ original draft. **SY:** writing ‐ review and editing. **TI:** visualization and supervision.

## CONFLICT OF INTEREST

The authors declare no conflicts of interest associated with this manuscript.

## Data Availability

The data that support the findings of this study are available from the corresponding author, Hiroshi Kobe, upon reasonable request.

## References

[1] Wu SG , Shih JY . Management of acquired resistance to EGFR TKI‐targeted therapy in advanced non‐small cell lung cancer. Mol Cancer. 2018;17(1):38. 29455650 10.1186/s12943-018-0777-1 PMC5817870

[2] Gushiken AC , Saharia KK , Baddley JW . Cryptococcosis. Infect Dis Clin North Am. 2021;35(2):493–514. 34016288 10.1016/j.idc.2021.03.012

[3] Taniwaki M , Yamasaki M , Ishikawa N , Kawamoto K , Hattori N . Pulmonary cryptococcosis mimicking lung cancer. Lancet Infect Dis. 2019;19(9):1033. 31478508 10.1016/S1473-3099(19)30278-6

[4] Ciochetto Z , Wainaina N , Graham MB , Corey A , Abid MB . Cryptococcal infection with ruxolitinib in primary myelofibrosis: a case report and literature review. Clin Case Rep. 2022;10(2):e05461. 35369391 10.1002/ccr3.5461 PMC8858788

[5] Qingsong L , Zhiji X , Liming P . Effects of ruxolitinib on infection in patients with myeloproliferative neoplasm: a meta‐analysis. Hematology. 2021;26(1):663–9. 34493151 10.1080/16078454.2021.1967256

[6] Zavala S , Baddley JW . Cryptococcosis. Semin Respir Crit Care Med. 2020;41(1):69–79. 32000285 10.1055/s-0039-3400280

[7] Ninomaru T , Hata A , Kokan C , Okada H , Tomimatsu H , Ishida J . Higher osimertinib introduction rate achieved by multiple repeated rebiopsy after acquired resistance to first/second generation EGFR‐TKIs. Thorac Cancer. 2021;12(6):746–51. 33475261 10.1111/1759-7714.13822 PMC7952804

